# Clinicopathological characteristics and outcome of primary sarcomatoid carcinoma of the gallbladder

**DOI:** 10.3389/fonc.2022.1009673

**Published:** 2022-09-27

**Authors:** Rui-Qi Zou, Hai-Jie Hu, Tian-Run Lv, Fei Liu, Wen-Jie Ma, Jun-Ke Wang, Yu-Shi Dai, Si-Qi Yang, Ya-Fei Hu, Fu-Yu Li

**Affiliations:** Division of Biliary Surgery, Department of General Surgery, West China Hospital, Sichuan University, Chengdu, China

**Keywords:** gallbladder, sarcomatoid carcinoma, curative resection, adjuvant chemotherapy, S-1

## Abstract

**Purpose:**

Our study aims to examine the clinicopathological features, disease progression, management, and outcomes of gallbladder sarcomatoid carcinoma (GBSC) patients.

**Methods:**

Between January 2000 and December 2020, 50 gallbladder cancer (GBC) patients who received surgical treatment and were pathologically verified as GBSC at our institution were enrolled. The clinical and pathological features and survival of these patients were retrospectively reviewed.

**Results:**

The median overall survival (OS) of GBSC patients was 14.5 months, and the 1-, 2- and 3-year OS rates were 68.0%, 32.0%, and 10.0%, respectively. The median progression-free survival (PFS) was 10.0 months, and the 1-, 2-, and 3-year PFS rates were 42.0%, 16.0%, and 2.0%, respectively. Patients who received radical resection had obviously better OS (18.0 *vs*. 7.0 months, P<0.001) and PFS (12.0 *vs*. 5.0 months, P<0.001) than those who underwent palliative resection. Multivariate analysis revealed that vascular invasion (P=0.033), curative operation (P<0.001) and postoperative chemotherapy (P=0.033) were independent risk factors for PFS. We further identified postoperative chemotherapy (P=0.010) and curative operation (P<0.001) as independent prognostic factors affecting the OS of GBSC patients. After curative surgery, patients who underwent S-1-based chemotherapy showed significantly longer recurrence-free survival (RFS) than those who underwent other chemotherapy regimens (20.0 *vs* 11.0 months, P=0.028).

**Conclusion:**

GBSC patients always have aggressive biological behaviors and remarkably poor prognoses. Most GBSC patients are diagnosed in advanced stages, and timely radical operation together with postoperative chemotherapy is important. S-1-based chemotherapy may be a selectively efficient regimen to prolong the survival of GBSC patients.

## Introduction

Gallbladder cancer (GBC) is the most common malignant tumor of the biliary tract and the sixth most common tumor among gastrointestinal cancers (1, 2). GBC is often detected at an advanced stage because most of them lack obvious symptoms in their early stages (3). GBC often presents aggressive characteristics such as local invasion, regional lymph node metastasis and distant metastases. The overall mean survival time for GBC patients is unsatisfactory, with an overall five-year survival of less than 5% (4–6). For patients with GBC, radical resection with negative margins remains the only curative treatment option (6–9).

Adenocarcinoma is the vast majority of GBC, accounting for approximately 80-90% (10). Other unusual subtypes include adenosquamous cell carcinoma, squamous cell carcinoma, neuroendocrine source tumors, undifferentiated carcinoma, mesenchymal tissue source tumors and so on (11, 12). The clinical and pathological characteristics, surgical management, therapeutic effect and prognosis may be different among these subgroups. Sarcomatoid carcinoma (SC) is a rare malignancy, with spindle cells similar to sarcomas but not being a distinct histological entity (13, 14). SC has been reported in several parts of the body, mainly developing in the kidneys, lungs, prostate, liver and so on (14–17). Gallbladder sarcomatoid carcinoma (GBSC) is one of the most unusual subtypes of gallbladder malignancy. Almost all GBSC patients were diagnosed according to the postoperative pathological examinations of the resected specimens. Compared with other patients with high-stage GBC, patients with GBSC often show aggressive progression and an unsatisfactory prognosis. To date, only a few reports of GBSC have been published, and the literature regarding these tumors mainly consists of case reports and small case series. Therefore, the clinical and pathological characteristics, surgical management and systematic therapy of GBSC are still at an initial and exploratory stage (18–21).

To define the behavior and prognosis of GBSC, in our study, relying on the largest sample size, we aimed to systematically examine the clinical and pathological features, disease progression, surgical management, adjuvant therapeutic effect and survival outcomes of GBSC patients.

## Methods

### Patient selection

We retrospectively reviewed all GBC patients diagnosed and treated at our institution (West China Medical Center, Sichuan University) between January 2000 and December 2020. The inclusion criteria and exclusion criteria were as follows. Inclusion criteria: (1) Patients with pathologically verified diagnosis of GBSC by paraffin sections; (2) Patients who underwent operations (including curative and palliative) in our institution. Exclusion criteria: (1) Preoperative radiotherapy or neoadjuvant chemotherapy was received; (2) Patients underwent reresection in other institutions after initial operation. Eventually, 50 resected GBSC cases were enrolled in our research for further analyses. Institutional Review Board approval was obtained for our study at West China Hospital.

### Data collection and follow-up

Retrospectively, we comprehensively collected clinical data of the 50 GBSC patients on demographics, including age, sex, height, weight and body mass index (BMI), and clinicopathological features, including gallstone coexistence, preoperative symptoms, tumor size, tumor location, American Joint Committee on Cancer (AJCC) tumor staging, liver parenchyma invasion, lymph node metastasis, distant metastasis, vascular invasion, perineural invasion, lymphovascular invasion and tumor differentiation degree. In addition, operation-related and postoperative variables, including preoperative serum parameters, surgical option, adjuvant chemotherapy regimen, postoperative radiotherapy and survival, were also obtained. The primary endpoint was overall survival (OS), defined as the time interval between operation and death or the last follow-up. Progression-free survival (PFS) was defined as the length of time from the operation to the latest follow-up without the disease worsening. Recurrence-free survival (RFS) was estimated as the length of time from the operation to the first documented recurrence. Progression and recurrence were confirmed by any new lesions detected by enhanced computed tomography (CT) or magnetic resonance imaging (MRI). All patients were strictly followed up with regular serum tumor markers and abdominal CT/MRI examinations in our institution.

### Pathological assessment

**Figure 1:**
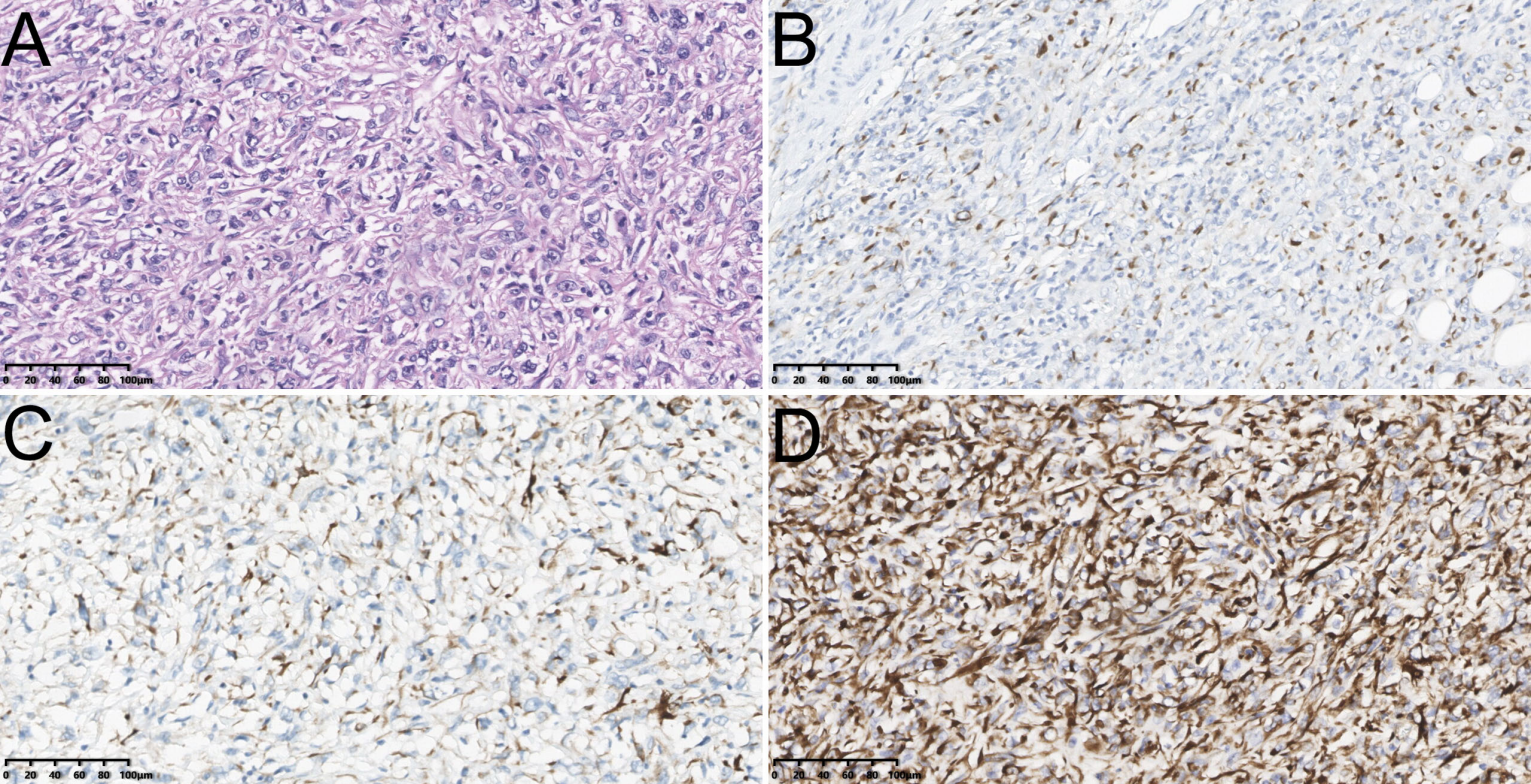
The hematoxylin-eosin (HE) staining of sarcomatoid carcinoma of gallbladder [Poor/undifferentiated, ×200, **(A)**]; The immunohistochemical staining of sarcomatoid carcinoma of gallbladder including Cytokeratin (CK)7 [Positive, ×200, **(B)**], Desmin [Negative, ×200, **(C)**] and Vimentin [Positive, ×200, **(D)**].

Our study used paraffin sections to determine the pathological evidence. Meanwhile, we verified all GBSC patients with two or more experienced pathologists in our institution before enrollment based on the World HealthOrganization (WHO) classification (**Figure 1**). According to the newly published (8th edition) AJCC classification of GBC, the tumors were staged (22).

### Statistical analysis

Numbers and percentages were used for categorical variables, while medians and ranges were used for continuous variables. An analysis of the survival curves was carried out using the Kaplan−Meier method, and the log-rank test was used to analyze further comparisons. We identified the potential prognostic factors using univariate and multivariate Cox proportional hazard regression models. SPSS 23.0 (IBM Corporation, Armonk, NY) was used for the statistical analyses. A P value less than 0.05 was considered statistically significant.

## Results

### Clinical characteristics

The clinical features of GBSC patients are shown in **Table 1**. Among the 50 GBSC patients, the male to female ratio was 20/30. The median age of GBSC patients was 62.0 (from 35 to 79) years. Abdominal discomfort (n=35, 70.0%), jaundice (n=10, 20.0%), weight loss (n=12, 24%) and pruritus (n=5, 10%) were common clinical symptoms. We also measured the preoperative serum levels of tumor biomarkers. Thirty-two (64%) patients showed an elevated carbohydrate antigen 19-9 (CA 19-9) level (median, 43.35 U/mL; normal value, <30 U/mL), while 12 (24%) patients showed an elevated carcinoembryonic antigen (CEA) level (median, 2.05 ng/mL; normal value, <5.0 ng/mL). All 50 GBSC patients showed space-occupying lesions in the gallbladder on computed tomography (CT) examination, the imaging findings of which were similar to those of conventional gallbladder adenocarcinoma patients.

**Table 1:** Clinical and pathological characteristics of the 50 resected cases of GBSC.

Variables	Median (range) or n (%)
Age, years, median (range)	62 (35-79)
Sex, male/female	20/30
Height, cm, median (range)	160 (145-170)
Weight, kg, median (range)	59 (40-72)
BMI, kg/m (^2^), median (range)	23.34 (14.53-28.89)
Gallstone, n (%)	31 (62)
Symptom, n (%)	
Abdominal discomfort	35 (70)
Weight loss	12 (24)
Jaundice	10 (20)
Pruritus	5 (10)
Tumor marker, median (range)	
CA19-9 (U/mL)	43.35 (4.12-1000.00)
CEA (ng/mL)	2.05 (0.29-195.00)
Liver function, median (range)	
TB (umol/L)	10.0 (4.90-190.0)
ALT (IU/L)	20.00 (5.00-143.00)
AST (IU/L)	20.00 (12.00-58.00)
Tumor size, cm, median (range)	6.5 (0.5-15)
Tumor location, n (%)	
Fundus	16 (32)
Body	13 (26)
Neck	8 (16)
Cystic duct	3 (6)
Multifocal	10 (20)
Perineural invasion n (%)	32 (64)
Lymphovascular invasion n (%)	24 (48)
Vascular invasion n (%)	
Portal vein	12 (24)
Hepatic artery	6 (12)
Liver parenchyma invasion n (%)	38 (76)
Lymph node metastasis n (%)	36 (72)
Distant metastasis n (%)	
Liver	3 (6)
Bone	1 (2)
Peritoneum	1 (2)
TNM staging n (%)	
I-II	7 (14.0)
III-IV	43 (86.0)
Differentiation degree n (%)	
Well/moderate	0 (0)
Poor/undifferentiated	50 (100)
Operation n (%)	
Curative	41 (82)
Palliative	9 (18)
Surgical option n (%)	
Cholecystectomy	46 (92)
Hepatectomy	40 (80)
Wedged resection	10 (20)
SIVb+SV resection	22 (44)
Right hemihepatectomy	8 (16)
Bile duct resection	16 (32)
Lymph node dissection	39 (80)
T tube drainage/Stent placement	4 (8)
Postoperative radiotherapy, n (%)	15 (30)
Postoperative chemotherapy, n (%)	
GX combination	4 (8)
GS combination	6 (12)
GP combination	4 (8)
AG combination	2 (4)
S-1 monotherapy	8 (16)
Capecitabine monotherapy	6 (12)

BMI, Body mass index; CA19-9, Cancer antigen 19-9; CEA, Carcinoembryonic antigen; TB, Total bilirubin; ALT, Alanine transaminase; AST, Aspartate aminotransferase; TNM, Tumor node metastasis; GBSC, Gallbladder sarcomatoid carcinoma.

### Pathological features

The pathological features of GBSC patients are shown in **Table 1**. In our study, all 50 GBSC patients underwent surgical treatment. There were 41 curative (82%) and 9 palliative (18.0%) intent operations. The median diameter of tumors was 6.5 cm (range, 0.5-15 cm). There were 43 (86%) patients who already had advanced cancer (III/IV stage) at the time of their initial diagnosis. All of the resected tumors showed poor/undifferentiated differentiation. Among the 50 resected patients, 32 (64%) had perineural invasion, 24 (48%) lymphovascular invasion, 14 (28%) vascular invasion, 38 (76%) liver parenchyma invasion and 36 (72%) lymph node metastasis. Liver metastasis, found in three patients (6.0%), was the most frequent site. Metastases to bone and peritoneum were observed in 1 (2.0%) patient each.

### OS and PFS of GBSC

**Figure 2:**
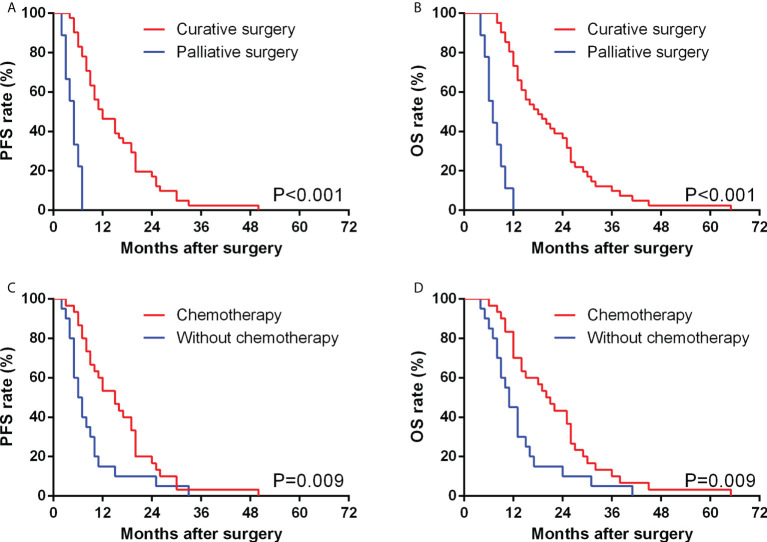
Comparison of PFS **(A)** and OS **(B)** between GBSC patients who underwent curative surgery and those who underwent palliative surgery by Kaplan−Meier analysis. Comparison of PFS **(C)** and OS **(D)** between GBSC patients who received postoperative chemotherapy and those who did not receive postoperative chemotherapy by Kaplan−Meier analysis.

**Figure 3:**
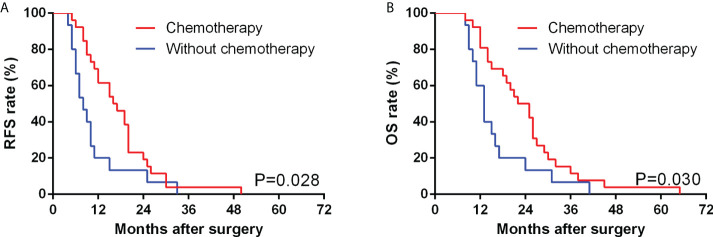
Comparison of RFS **(A)** and OS **(B)** between GBSC patients who underwent curative surgery with and without chemotherapy by Kaplan−Meier analysis. (Among the GBSC patients who underwent curative surgery, 26 received postoperative chemotherapy, while 15 of them received strictly following up alone without postoperative chemotherapy).

The median PFS time of 50 GBSC patients was 10.0 months, with 1-, 2-, and 3-year PFS rates of 42.0%, 16.0%, and 2.0%, respectively. In GBSC patients, the median survival time was 14.5 months, and the 1-, 2-, and 3-year survival rates were 68.0%, 32.0%, and 10.0%, respectively. (**Figures 2**, **3**)

### Prognostic factors for PFS in the GBSC cohort

Based on univariate analysis, vascular invasion (P=0.002), TNM staging (P = 0.021), postoperative chemotherapy (P=0.014), curative operation (P<0.001), liver parenchyma invasion (P=0.011) and perineural invasion (P=0.034) were significant predictors of PFS in patients with GBSC. Multivariate analysis further identified vascular invasion (P=0.033), curative/palliative operation (P<0.001) and postoperative chemotherapy (P=0.033) as independent prognostic factors affecting the PFS of GBSC patients (**Table 2**).

**Table 2:** Univariate and multivariate analysis of PFS for patients with GBSC.

Variables	No. of patientsn(%)	median PFS, months	Survival rate(%)		P value	Multivariate analysis	
			1 year	3 year		HR (95% CI)	P value
Age					0.399		
>60	27	10.0	37.0	0.0			
≤60	23	10.0	47.8	4.3			
Sex					0.944		
Male	20	11.0	45.0	0.0			
Female	30	9.5	40.0	3.3			
BMI					0.763		
>23	28	10.0	35.7	3.6			
≤23	22	12.0	50.0	0.0			
Gallstone					0.208		
Yes	31	11.0	45.2	3.2			
No	19	8.0	36.8	0.0			
Preoperative CA19-9 level					0.124		
>30U/ml	32	10.0	37.5	0.0			
≤30U/ml	18	11.5	50.0	5.6			
Preoperative CEA level					0.287		
>5ng/ml	12	8.5	41.7	8.3			
≤5ng/m	38	10.0	42.1	0.0			
Tumor size					0.209		
>5.0cm	31	9.0	35.5	0.0			
≤5.0cm	19	12.0	52.6	5.3			
Perineural invasion					**0.034**	1.086 (0.525-2.245)	0.824
Yes	32	7.5	31.3	0.0			
No	18	18.0	61.1	5.6			
Lymphovascular invasion					0.093		
Yes	24	10.0	37.5	0.0			
No	26	10.0	46.2	3.8			
Vascular invasion					**0.002**	2.284 (1.1067-4.888)	**0.033**
Yes	14	7.0	14.3	0.0			
No	36	12.0	52.8	2.8			
Liver parenchyma invasion					**0.011**	1.617 (0.601-4.354)	0.341
Yes	38	8.0	28.9	0.0			
No	12	20.0	83.3	8.3			
Lymph node metastasis					0.169		
Yes	36	9.0	33.3	0.0			
No	14	13.5	64.3	7.1			
TNM staging					**0.021**	1.121 (0.317-3.969)	0.860
I-II	7	20.0	85.7	14.3			
III-IV	43	9.0	34.9	0.0			
Operation					**<0.001**	0.133 (0.049-0.359)	**<0.001**
Curative	41	12.0	51.0	2.4			
Palliative	9	5.0	0.0	0.0			
Chemotherapy					**0.014**	0.484 (0.248-0.944)	**0.033**
Yes	30	15.0	60.0	3.3			
No	20	6.5	15.0	0.0			
Radiotherapy					0.363		
Yes	15	11.0	46.7	6.7			
No	35	9.0	40.0	0.0			

BMI, Body mass index; CA19-9, Cancer antigen 19-9; CEA, Carcinoembryonic antigen; TNM, Tumor node metastasis; GBSC, Gallbladder sarcomatoid carcinoma; CI, Confidence interval; HR, Hazard ratio; PFS, Progression-free survival.

Bold mark the factors that P<0.05 in the univariate and multivariate analyses.

### Prognostic factors for OS in the GBSC cohort

Based on univariate analysis, vascular invasion (P=0.001), TNM staging (P=0.025), liver parenchyma invasion (P=0.019), postoperative chemotherapy (P=0.013) and curative/palliative operation (P<0.001) were significant predictors of PFS in patients with GBSC. Multivariate analysis further identified postoperative chemotherapy (P=0.010) and curative operation (P<0.001) as independent prognostic predictors for the OS of GBSC patients (**Table 3**).

**Table 3:** Univariate and multivariate analysis of OS for patients with GBSC.

Variables	No. of patientsn (%)	median OS, months	Survival rate(%)		P value	Multivariate analysis	
			1 year	3 year		HR (95% CI)	P value
Age					0.415		
>60	27	14.0	63.0	7.4			
≤60	23	15.0	73.9	13.0			
Sex					0.832		
Male	20	15.0	80.0	15.0			
Female	30	13.0	60.0	6.7			
BMI					0.954		
>23	28	13.5	71.4	14.3			
≤23	22	17.0	63.6	4.5			
Gallstone					0.161		
Yes	31	16.0	74.0	9.7			
No	19	12.0	57.9	10.5			
Preoperative CA19-9 level					0.133		
>30U/ml	32	14.0	71.9	3.1			
≤30U/ml	18	18.0	61.1	22.2			
Preoperative CEA level					0.245		
>5ng/ml	12	13.5	66.7	16.7			
≤5ng/m	38	14.5	68.4	7.9			
Tumor size					0.135		
>5.0cm	31	13.0	64.5	6.5			
≤5.0cm	19	18.0	73.7	15.8			
Perineural invasion					0.081		
Yes	32	12.5	56.3	6.3			
No	18	23.5	88.9	16.7			
Lymphovascular invasion					0.109		
Yes	24	14.0	66.7	0.0			
No	26	15.5	69.2	19.2			
Vascular invasion					**0.001**	2.056 (0.952-4.441)	0.067
Yes	14	11.0	42.9	0.0			
No	36	19.0	77.8	13.9			
Liver parenchyma invasion					**0.019**	1.397 (0.528-3.700)	0.501
Yes	38	12.5	60.5	7.9			
No	12	26.0	91.7	16.7			
Lymph node metastasis					0.174		
Yes	36	13.0	61.1	8.3			
No	14	19.5	85.7	14.3			
TNM staging					**0.025**	1.292 (0.372-4.485)	0.686
I-II	7	29.0	100.0	28.6			
III-IV	43	13.0	62.8	7.0			
Operation					**<0.001**	0.102 (0.038-0.274)	**<0.001**
Curative	41	18.0	80.5	12.2			
Palliative	9	7.0	11.1	0.0			
Chemotherapy					**0.013**	0.439 (0.234-0.825)	**0.010**
Yes	30	20.5	83.3	13.3			
No	20	11.0	45.0	5.0			
Radiotherapy					0.197		
Yes	15	16.0	86.7	20.0			
No	35	13.5	60.0	5.7			

BMI, Body mass index; CA19-9, Cancer antigen 19-9; CEA, Carcinoembryonic antigen; TNM, Tumor node metastasis; GBSC, Gallbladder sarcomatoid carcinoma; CI, Confidence interval; HR, Hazard ratio; OS, Overall survival.

Bold mark the factors that P<0.05 in the univariate and multivariate analyses.

### Chemotherapy

In our study, 30 (60.0%) patients underwent systematic postoperative chemotherapy in total, including oral and intravenous approaches. Among them, 16 (53.3%) patients underwent gemcitabine-based chemotherapy, in which 4 (13.3%) underwent the GX regimen (Gemcitabine + Xeloda (capecitabine tablets)), 6 (20.0%) underwent the GS regimen (Gemcitabine + S-1 (tegafur, gimeracil and oteracil potassium capsules)), 4 (13.3%) underwent the GP regimen (Gemcitabine + Cis-platinum) and 2 (6.7%) underwent the AG regimen (Gemcitabine + Albumin-bound paclitaxel). Fourteen (46.7%) patients underwent oral chemotherapy, including 8 (26.7%) with S-1 monotherapy and 6 (20%) with capecitabine monotherapy. Compared with patients without postoperative chemotherapy, those patients who underwent systematic chemotherapy showed a significantly better PFS (15.0 *vs* 6.5 months, P=0.009) and OS (20.5 *vs* 11.0 months, P=0.009).

**Figure 4:**
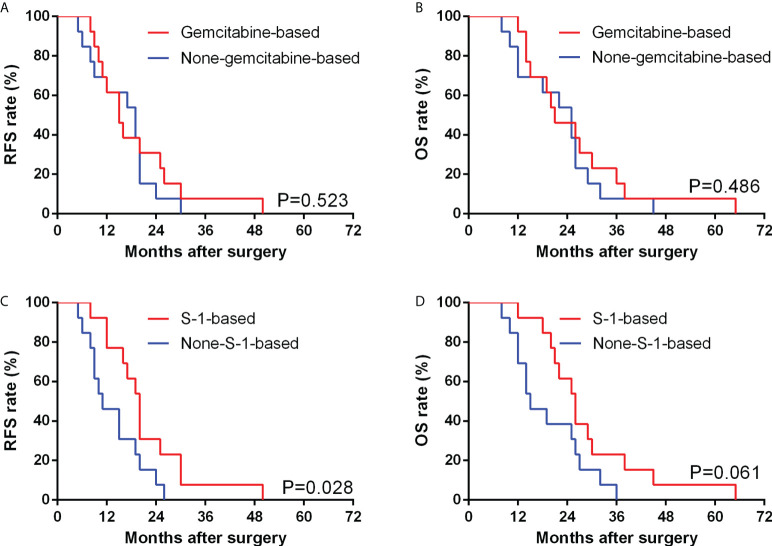
Comparison of RFS **(A)** and OS **(B)** between GBSC patients who underwent curative surgery with gemcitabine-based chemotherapy and non-gemcitabine-based chemotherapy by Kaplan−Meier analysis; Comparison of RFS **(C)** and OS **(D)** between GBSC patients who underwent curative surgery with S-1-based chemotherapy and non-S-1-based chemotherapy by Kaplan−Meier analysis. (Among the GBSC patients who underwent curative surgery, 26 received postoperative chemotherapy, including 3 of GX combination, 3 of GP combination, 2 of AG combination, 5 of GS combination, 8 of S-1 monotherapy and 5 of Xeloda monotherapy).

In patients after curative operation, those after gemcitabine-based chemotherapy (defined as the chemotherapy regimens containing the gemcitabine in our study, includes GX combination, GS combination, GP combination and AG combination) showed no significant difference in median RFS (P=0.523) and OS (P=0.486) compared with counterparts with other chemotherapy regimens (**Figure 4**). We further compared the RFS and OS between patients whether underwent S-1-based chemotherapy (defined as the chemotherapy regimens containing the S-1 in our study, includes GS combination and S-1 monotherapy) or not, and found that patients who underwent S-1-based chemotherapy showed significantly longer RFS than those who underwent other chemotherapy regimens (20.0 *vs* 11.0 months, P=0.028), although there was no significant difference between the OS of the two groups (26.0 *vs* 15.0 months, P=0.061) (**Figure 4**). Among 13 patients undergoing S-1-based chemotherapy after curative operation, 10 (76.9%) patients showed normal CA 19-9 levels, while 8 of 13 (61.5%) patients who underwent non-S-1-based chemotherapy presented an elevated CA19-9 level.

## Discussion

Due to their rarity, GBSCs have unusually been encountered in clinical practice. Therefore, the literature regarding these tumors mainly consists of case reports and small case series. In our analysis, 1395 GBC patients have been screened for GBSC, and the approximate incidence rate of GBSC in GBC patients were 3.6%, which was similar to the 4.3% in prior literature report (23). The clinical and pathological features and outcomes of GBSC patients are largely unknown. GBSC has a high malignant potential with aggressive progression and a high rate of systemic metastasis, even in its early stages. Clinically, GBSC presents nonspecifically, similar to conventional adenocarcinoma. Thus, the majority of the disease was diagnosed at an advanced stage.

In general, radical surgical resection with negative surgical width remains the only curative therapy for GBC (9). A standard surgical treatment strategy has not yet been determined for advanced GBSC, but prior studies have recommended radical cholecystectomy with lymph node dissection and liver resection. In a previous study involving six patients with GBSC, three patients underwent radical resection (23). The longest survival was five months in this case series, while the median was 2.5 months. The GBSC patients who underwent curative treatment did not show significantly longer OS than those who underwent palliative treatment. Thus, the author thought that the prognosis of GBSC did not depend on tumor node metastasis (TNM) stage and was extremely poor even in patients who received radical resection with or without postoperative adjuvant chemotherapy. In our study, surgical treatment was carried out in all 50 GBSC patients, including radical and palliative operations, and all TNM stage III patients received radical cholecystectomy. However, GBSC patients who underwent radical resection had a significantly better OS than those who underwent palliative surgery, which was different from the results of previous studies.

Prior publications have indicated that vascular invasions are more common in SC than in conventional adenocarcinoma (17, 24–26). Our study came to the same result, and we thought it may be ascribed to the poor differentiation degree and high invasive tendency of these tumors. Among our 14 patients with vascular invasion, 12 had portal vein invasion, and 6 had hepatic artery invasion, most of which had early recurrence after curative surgery. Even though portal vein or hepatic artery resection and reconstruction had been performed, the postoperative survival still seemed unsatisfactory. Our multivariate analysis further suggested that vascular invasion was strongly associated with poorer RFS in GBSC. We speculated that the sarcomatous component of GBSC may be responsible for the aggressive behaviors and its earlier recurrence and metastasis.

Various adjuvant chemotherapy strategies have been used in GBC patients following radical resection; however, the benefit of adjuvant chemotherapy has always been questioned, especially for GBSC (11). In a prior case series, one of seven GBSC cases underwent systematic adjuvant therapy, including GX regimen chemotherapy and radiotherapy, achieving a PFS of 12 months and OS of 15 months, which were the longest reported survival to date in such patients (21). In our study, we retrospectively collected data from 50 GBSC patients, including 30 patients who received adjuvant chemotherapy after surgical resection, and the results showed that these patients had a longer median PFS and OS than those without adjuvant chemotherapy. Similar results were also found among patients who underwent radical operation. Hence, in consideration of aggressive biological behaviors, unsatisfactory survival rates and high recurrence rates of GBSC, we recommended that even after radical resection, postoperative adjuvant chemotherapy was equally important.

Since there is still no standard chemotherapy regimen for GBSC, we commonly use similar regimens for conventional gallbladder adenocarcinoma. According to the new NCCN guidelines, gemcitabine monotherapy or in combination with cisplatin or capecitabine, capecitabine monotherapy or in combination with cisplatin or oxaliplatin, and 5-fluorouracil are recommended chemotherapy regimens (11). In our study, 60% of patients underwent postoperative chemotherapy. Among them, 16 patients underwent gemcitabine-based chemotherapy, while 14 patients underwent non-gemcitabine-based chemotherapy. After excluding those without curative surgery, we found that there was no significant difference regarding the RFS or OS between patients with gemcitabine-based chemotherapy and those with non-gemcitabine-based chemotherapy. Thus, we speculate that gemcitabine-based chemotherapy might not remarkably prolong the survival outcomes of patients with GBSC.

Hence, to explore why GBSC patients after chemotherapy had better survival outcomes, we further divided patients into S-1-based chemotherapy (n=14) and non-S-1-based chemotherapy (n=16). After excluding those without curative surgery, we found that patients with S-1-based chemotherapy showed better RFS when compared with the non-S-1-based group, and patients with S-1-based chemotherapy benefited from a median RFS of 9.0 months. Further analysis showed that patients with S-1-based chemotherapy could have a nonsignificant OS benefit when compared to the non-S-1-based group. Based on these findings, we think S-1-based chemotherapy could serve as a potential effective regimen for GBSC patients.

S-1, composed of tegafur, gimeracil and oteracil potassium, is a second-generation fluorouracil chemotherapeutic agent. S-1 has been widely used for the treatment of malignancy in Japan, including gastrointestinal, pancreatic, lung cancer and so on (27–30). Because of the few adverse reactions and short half-life of the tegafur, gimeracil and oteracil potassium capsule, it is quick, easy and safe for us to administer, and it avoids pain and side effects associated with intravenous fluids. Meanwhile, oral administration also improved patient compliance and is less likely to develop drug resistance (31). Several studies regarding S-1 chemotherapy have been ongoing, and most of the publications were from Japan. In prior research that enrolled 186 node-positive perihilar cholangiocarcinoma patients, the RFS was longer in the S-1 group than in the gemcitabine-based group (median, 24.4 months *vs* 14.9 months; P=0.044) among patients who underwent postoperative adjuvant therapy (32). Another author reported that in unresectable biliary tract cancer (243 patients total), S-1-based and gemcitabine-based chemotherapy showed similar efficacy in terms of response rate (RR), disease control rate (DCR), PFS and OS (33, 34). In our current research, 13 patients underwent chemotherapy containing S-1 also presented improved survival. According to our result, we thought that applying S-1-based chemotherapy to postoperative GBSC may be a selectively efficient regimen to prolong the survival. Of course, further studies with larger sample sizes and molecular data are needed to verify our conclusion.

CA 19-9 has been widely used in the early diagnosis and prognostic prediction of GBC (35, 36). Elevated serum CA 19-9 levels could be suggestive of GBC occurrence and progression. Prior studies showed that an elevated CA 19-9 was accompanied by an increased risk of recurrence of bile duct tumors (37–39). Interestingly, we analyzed the CA 19-9 levels of GBSC patients after a year of radical operation and found that those patients who underwent S-1-based chemotherapy had more stable CA 19-9 levels than those without chemotherapy or with other chemotherapy regimens. Among patients undergoing S-1-based chemotherapy after curative operation, 76.9% of patients showed normal CA 19-9 levels, which is obviously better than those who underwent non-S-1-based chemotherapy. This also supports our results that patients after S-1-based chemotherapy may have longer RFS than their counterparts with other chemotherapy regimens.

The retrospective study method and limited sample size from a single institution were the major limitations of the study. Further multicenter and prospective studies with larger sample sizes are urgently needed to support our conclusions. Meanwhile, our study lacks molecular data, which is another limitation. Further research is warranted to identify specific molecular features correlated with the survival of GBSC patients.

In conclusion, our study systematically demonstrates the clinical and pathological features of GBSC. Furthermore, we explored the correlation between these features and the prognoses of GBSC patients. GBSC showed significant aggressive biological behaviors, together with more advanced clinicopathological features and obviously inferior prognosis. Those patients who underwent systematic chemotherapy showed significantly better prognoses than those without postoperative chemotherapy. Interestingly, we found that among patients who underwent curative surgery, those who underwent S-1-based chemotherapy showed a significantly longer RFS than their counterparts who underwent other chemotherapy regimens. We thought S-1-based chemotherapy might be a selectively efficient regimen to prolong the prognoses of postoperative GBSC patients.

## Data availability statement

The raw data supporting the conclusions of this article will be made available by the authors, without undue reservation.

## Ethics statement

The study protocol was approved by the ethics committee review board of Sichuan University. Informed consent was obtained from all patients for surgical treatment.

## Author contributions

R-QZ and H-JH contributed equally to the manuscript and were the first co-authors. R-QZ and H-JH contributed to data acquisition and drafted the manuscript. T-RL, FL, W-JM, J-KW, Y-SD, S-QY, and Y-FH contributed to data acquisition. F-YL contributed to the study design and revision of the manuscript. All authors contributed to the article and approved the submitted version.

## Funding

Supported by 1.3.5 project for disciplines of excellence, West China Hospital, Sichuan University (ZYJC21046); 1.3.5 project for disciplines of excellence-Clinical Research Incubation Project, West China Hospital, Sichuan University (2021HXFH001); Sichuan Science and Technology Program (2021YJ0132, 2021YFS0100); The fellowship of China Postdoctoral Science Foundation (2021M692277); Sichuan University-Zigong School-local Cooperation project (2021CDZG-23); The Fundamental Research Funds for the Central Universities (2019SCUH0021); Science and Technology project of the Health planning committee of Sichuan (21PJ046); Post-Doctor Research Project, West China Hospital, Sichuan University (2021HXBH127).

## Conflict of interest

The reviewer SW declared a shared affiliation with the authors to the handling editor at the time of review.

The authors declare that the research was conducted in the absence of any commercial or financial relationships that could be construed as a potential conflict of interest.

## Publisher’s note

All claims expressed in this article are solely those of the authors and do not necessarily represent those of their affiliated organizations, or those of the publisher, the editors and the reviewers. Any product that may be evaluated in this article, or claim that may be made by its manufacturer, is not guaranteed or endorsed by the publisher.
